# An Extract-Transform-Load Process Design for the Incremental Loading of German Real-World Data Based on FHIR and OMOP CDM: Algorithm Development and Validation

**DOI:** 10.2196/47310

**Published:** 2023-08-21

**Authors:** Elisa Henke, Yuan Peng, Ines Reinecke, Michéle Zoch, Martin Sedlmayr, Franziska Bathelt

**Affiliations:** 1 Institute for Medical Informatics and Biometry, Carl Gustav Carus Faculty of Medicine, Technische Universität Dresden, Dresden, Saxony, Germany

**Keywords:** ETL, incremental loading, OMOP CDM, FHIR, interoperability, Extract-Transform-Load, Observational Medical Outcomes Partnership Common Data Model, Fast Healthcare Interoperability Resources

## Abstract

**Background:**

In the Medical Informatics in Research and Care in University Medicine (MIRACUM) consortium, an IT-based clinical trial recruitment support system was developed based on the Observational Medical Outcomes Partnership (OMOP) Common Data Model (CDM). Currently, OMOP CDM is populated with German Fast Healthcare Interoperability Resources (FHIR) using an Extract-Transform-Load (ETL) process, which was designed as a bulk load. However, the computational effort that comes with an everyday full load is not efficient for daily recruitment.

**Objective:**

The aim of this study is to extend our existing ETL process with the option of incremental loading to efficiently support daily updated data.

**Methods:**

Based on our existing bulk ETL process, we performed an analysis to determine the requirements of incremental loading. Furthermore, a literature review was conducted to identify adaptable approaches. Based on this, we implemented three methods to integrate incremental loading into our ETL process. Lastly, a test suite was defined to evaluate the incremental loading for data correctness and performance compared to bulk loading.

**Results:**

The resulting ETL process supports bulk and incremental loading. Performance tests show that the incremental load took 87.5% less execution time than the bulk load (2.12 min compared to 17.07 min) related to changes of 1 day, while no data differences occurred in OMOP CDM.

**Conclusions:**

Since incremental loading is more efficient than a daily bulk load and both loading options result in the same amount of data, we recommend using bulk load for an initial load and switching to incremental load for daily updates. The resulting incremental ETL logic can be applied internationally since it is not restricted to German FHIR profiles.

## Introduction

### Background and Significance

Randomized controlled trials are the gold standard to “measure the effectiveness of a new intervention or treatment” [1]. However, randomized controlled trials are limited regarding the representative number of persons included and, therefore, restricted in their external generalizability. To gain more unbiased evidence, observational studies focus on real-world data from large heterogeneous populations.

To support observational research, we already provide a transferable Extract-Transform-Load (ETL) process [2] to transform German real-world data to the Observational Medical Outcomes Partnership (OMOP) Common Data Model (CDM) [3] provided by Observational Health Data Sciences and Informatics (OHDSI) [4], which supports the possibilities for multicentric and even international studies. Due to the heterogeneity of the structure and content of the data from the data integration centers within the Medical Informatics Initiative Germany (MI-I) [5], the Health Level 7 (HL7) [6] Fast Healthcare Interoperability Resources (FHIR) communication standard was specified among all German university hospitals. Consequently, we used FHIR as the source for our ETL process. The FHIR specification is given by the core data set of the MI-I [7]. FHIR resources can be read from an FHIR Gateway [8] (PostgreSQL database) or FHIR Server (eg, HAPI [9] or Blaze [10]). As the target of our ETL process, we used OMOP CDM v5.3.1 [11]. The implementation of the ETL process was done using the open source framework Java SpringBatch [12]. Our ETL process has been implemented in accordance with the default assumption as described in the Book of OHDSI [13], where the OHDSI community defines the ETL process as a full load to transfer data from source to target systems. This approach is efficient for a dedicated study where data gets loaded once without any update afterward but inefficient if it comes to the need for updated data on a daily basis.

Latter is the case for the developments around the improvement and support of the recruitment process for clinical trials, which the Medical Informatics in Research and Care in University Medicine (MIRACUM) [14] consortium, as part of the MI-I funded by the German Federal Ministry of Education and Research, is working on. In this context, an IT-based clinical trial recruitment support system (CTRSS) based on OMOP CDM was implemented [15]. The CTRSS consists of a screening list for recruitment teams that provides potential candidates for clinical trials updated on a daily base. To enable the CTRSS to provide recruitment proposals, it is necessary to transform the data in FHIR format at each site from the 10 MIRACUM data integration centers into the standardized format of OMOP CDM. The procession of FHIR resources to OMOP CDM through our ETL process has already been successfully tested and integrated at all 10 German university hospitals of the MIRACUM consortium.

So far, our ETL process is restricted to a bulk load of FHIR resources to OMOP CDM. This implied that all FHIR resources are read from the source. To enable the CTRSS to provide daily recruitment proposals, our ETL process has to be executed every day as a full load. However, an everyday full load is not efficient because often only a small amount of source data has changed during loading periods, which results in unnecessary long execution times considering a full load for daily executions. Consequently, the computational effort that comes with the daily execution of the bulk load is not efficient in the context of the CTRSS.

Thus, a new approach is needed to only process FHIR resources that were created, updated, or deleted (CUD) since the last execution of the ETL process once an initial load has been executed. This loading option is known as incremental loading.

### Objective

To keep the bulk load option for dedicated studies and still be performant toward daily changes in the source data, a combination of bulk load and incremental load is needed. To reduce the additional effort in implementing a second independent ETL process for incremental loading, it is our aim to extend our existing ETL process with the option of incremental loading. During our research, we focused on the following four research questions:

1. What requirements need to be considered when integrating incremental loading into our existing ETL process design?
2. What approaches already exist for incremental ETL processes?
3. How can the identified requirements from research question 1 be implemented in our existing ETL process design?
4. Does incremental loading provide an advantage over daily bulk loading?

## Methods

### Analysis of the Existing ETL FHIR-to-OMOP Process

To determine the requirements for integrating incremental loading into our existing ETL process design, we performed an impact analysis focusing on the whole ETL process as well as, in more detail, the three main components of it, namely, Reader, Processor, and Writer as presented by Peng et al [2]. Regarding the whole ETL process, the following 3 requirements were needed:

- Requirement A: It is necessary to provide the user with the ability to distinguish between bulk loading and incremental loading.
- Requirement B: For incremental loading, it is further essential that the Reader of the ETL process is able to detect changes in the source system and reads only CUD-FHIR resources on a daily basis.
- Requirement C: During the processing of updated and deleted FHIR resources, duplicates and obsolete data should be avoided in OMOP CDM to guarantee data correctness.

Considering the semantic mapping from FHIR MI-I Core Data Set (CDS) to OMOP CDM and the Writer of the ETL process as described by Peng et al [2], incremental loading has no impact on both. In summary, incremental loading requires an adjustment of the implementation of the Reader and Processor.

### Literature Review

To identify approaches that might be adaptable to our existing ETL design and fulfill the 3 requirements in the previous section, we conducted a first literature review on July 14, 2021; a second one on November 28, 2022; and a third one on February 22, 2023 (Multimedia Appendices 1, 2, and 3). Table 1 includes the search strings and the number of results for three literature databases.

**Table 1.** Literature review: database, search string, and number of results.

Database	Search string	Results, n
PubMed	All fields: (incremental) AND ((etl) OR (extract transform load))	7
IEEE Xplore	((“All Metadata”: incremental) AND (“All Metadata”: etl OR “All Metadata”: extract transform load))	15
Web of Science	ALL=(incremental) AND (ALL=(etl) OR ALL=(extract transform load))	46

We included only articles from 2011 to 2022 in English. After removing duplicates, 51 items were left. These were screened independently by two authors (EH and MZ). Through the title and abstract screening, we identified 12 relevant articles. After the screening of the full texts, we included 8 articles within our research. Reasons for excluding the other articles were other meanings of the abbreviation “ETL,” ETL tools without regard to theoretical approaches of incremental loads, focus on application instead of ETL process and theoretical approach, and quality and error handling without focus on a theoretical approach.

Only 2 of the 8 articles addressed ETL processes for loading patient data into OMOP CDM. Lynch et al [16] introduced an approach for incremental transformation from the data warehouse to OMOP CDM to prevent incremental load errors. They suggest basing the development on a quality assurance process regarding the data quality framework by Kahn et al [17]. Furthermore, they generated ETL batch tracking ids for each record of data during the transformation to OMOP CDM. For 1:1 mappings, they created custom columns in the standardized OMOP CDM tables, and for 1:n or n:1 mappings, they used a parallel mapping table to store the ETL batch id and a link to the corresponding record in OMOP CDM. Lenert et al [18] describe an automated transformation of clinical data into two CDMs (OMOP and PCORnet database) by using FHIR. Therefore, they use the so-called subscriptions of FHIR resources. These subscriptions trigger a function to create a copy of the FHIR resource and its transmission into another system whenever an FHIR resource is created or updated.

Despite OMOP CDM being the target database, the literature search revealed different concepts for incremental ETL itself. Kathiravelu et al [19] described the caching of new or updated data in a temporary table. Of the 8 articles, 7 described various methods for incremental updates, particularly focusing on change data capture (CDC). All describe different categories of CDC, like timestamp-based, audit column–based, trigger-based, log-based, application programming interface–based, and data-based snapshots [16,18,20-24]: (1) Lynch et al [16] and (2) Lenert et al [18] focused on triggers; (3) Wen [20] focused on timestamps and triggers; (4) Thulasiram and Ramaiah [21] and (5) Sun [22] focused on timestamps; (6) Hu and Dessloch [23] focused on timestamps, audit columns, logs, triggers, and snapshots; and (7) Wei Du and Zou [24] focused on snapshots and MapReduce.

In summary, the literature review revealed adaptable approaches, which can be applied for the implementation of requirements B and C. However, no approaches could be found in the literature for requirement A. For this reason, we have to define a new method to enable both bulk and incremental loading in one ETL process. The concrete integration of the approaches into our existing ETL design is described in more detail in the following sections.

### Incremental ETL Process Design

#### Enabling Both Bulk and Incremental Loading

For the specification, if the ETL process should be executed as bulk or incremental load, we added a new Boolean parameter in the configuration file of the ETL process called APP_BULKLOAD_ENABLED. According to the desired loading option, the parameter has to be adjusted before executing the ETL process, with “true” results in a bulk load and “false” results in an incremental load. During the execution of the ETL process, this parameter is further taken into account for the Reader and Processor of the ETL process [2] to distinguish between the needs of bulk and incremental load (eg, to ensure that the OMOP CDM database is not emptied at the beginning of the ETL process execution during an incremental load).

#### Focusing on CUD-FHIR Resources Since the Last ETL Execution

Our purpose of incremental loading was to focus only on CUD-FHIR resources since the last time the ETL process was executed (whether as bulk or incremental load). Consequently, the ETL process for incremental load has to filter only CUD-FHIR resources from the source. The literature research showed that there are various CDC approaches to detect changes in the source. In our case, FHIR resources in the FHIR Gateway and FHIR Server contain metadata, such as a timestamp indicating when an FHIR resource was created, updated, or deleted in the source (FHIR Gateway: column last_updated_at; FHIR Server: meta.lastUpdated). That is why we used the timestamp-based CDC approach to filter FHIR resources, which have a timestamp specification after the last ETL execution time.

To ensure the filtering for the incremental load, we added two new parameters in the configuration file of the ETL process: DATA_BEGINDATE and DATA_ENDDATE. Both parameters have to be adjusted before executing the ETL process as incremental load. During the execution, the ETL process takes these two parameters into account and only reads FHIR resources from the source that has a metadata timestamp specification that is in [DATA_BEGINDATE, DATA_ENDDATE].

#### Guarantee Data Correctness in OMOP CDM

To avoid duplicates in OMOP CDM when processing updated and deleted FHIR resources, their existence in OMOP CDM has to be checked during the processing. The FHIR resources themselves do not have a flag that indicates whether they are new or have been changed. Only deleted FHIR resources can be identified by a specific flag in the metadata. To assess the existence of FHIR resources in OMOP CDM, a comparison of the data of the read FHIR resources with the data already available in OMOP CDM has to be done.

The literature research showed an approach to generate a unique tracking id per source data during the transformation process and its storage in OMOP CDM [16]. We decided against the approach of generating an additional id because FHIR resources already contain two identifying FHIR elements themselves: id and identifier. The id represents the logical id of the resource per resource type while the identifier specifies an identifier that is part of the source data. Both FHIR elements allow the unique identification of an FHIR resource per resource type. However, the standardized OMOP CDM tables do not provide the possibility to store this information from FHIR. Furthermore, OMOP CDM has its own primary keys for each record in a table independent of the id and identifier used in FHIR. Consequently, after transforming FHIR resources to OMOP CDM, the identifying data from FHIR resources will be lost.

To solve this problem, we need to store the mapping between the id and identifier used in FHIR with the id used in OMOP CDM. Due to the fact that the id of an FHIR resource is only unique per resource type and one FHIR resource can be stored in OMOP CDM in multiple tables, we additionally have to specify the resource type. As mentioned above, Lynch et al [16] presented an approach to store the mapping between tracking ids for source records and ids used in OMOP CDM by using a mapping table and custom columns in OMOP CDM. We have slightly customized this approach and adapted it into our ETL design. Contrary to the use of both mapping tables and custom columns, we considered each approach separately.

Our first approach uses mapping tables for each FHIR resource type in a separate schema in OMOP CDM. With this approach, the Writer of the ETL process has to fill additional mapping tables beside the standardized tables in OMOP CDM. Our second approach focuses on two new columns in the standardized tables in OMOP CDM called “fhir_logical_id” and “fhir_identifier.” These columns store the id and identifier of the FHIR resource. Furthermore, we appended an abbreviation of the resource type as a prefix to the id and identifier of FHIR (eg, “med-” for Medication, “mea-” for MedicationAdministration, or “mes-” for MedicationStatement FHIR resources). In consequence, the combination of the prefix with the id and identifier and its storage in OMOP CDM enables the unique identification of FHIR resources in OMOP CDM. Since the mapping tables and two new columns are required exclusively for the ETL process, the analysis of data across multiple OMOP CDM databases is not affected.

**Figure 1.**
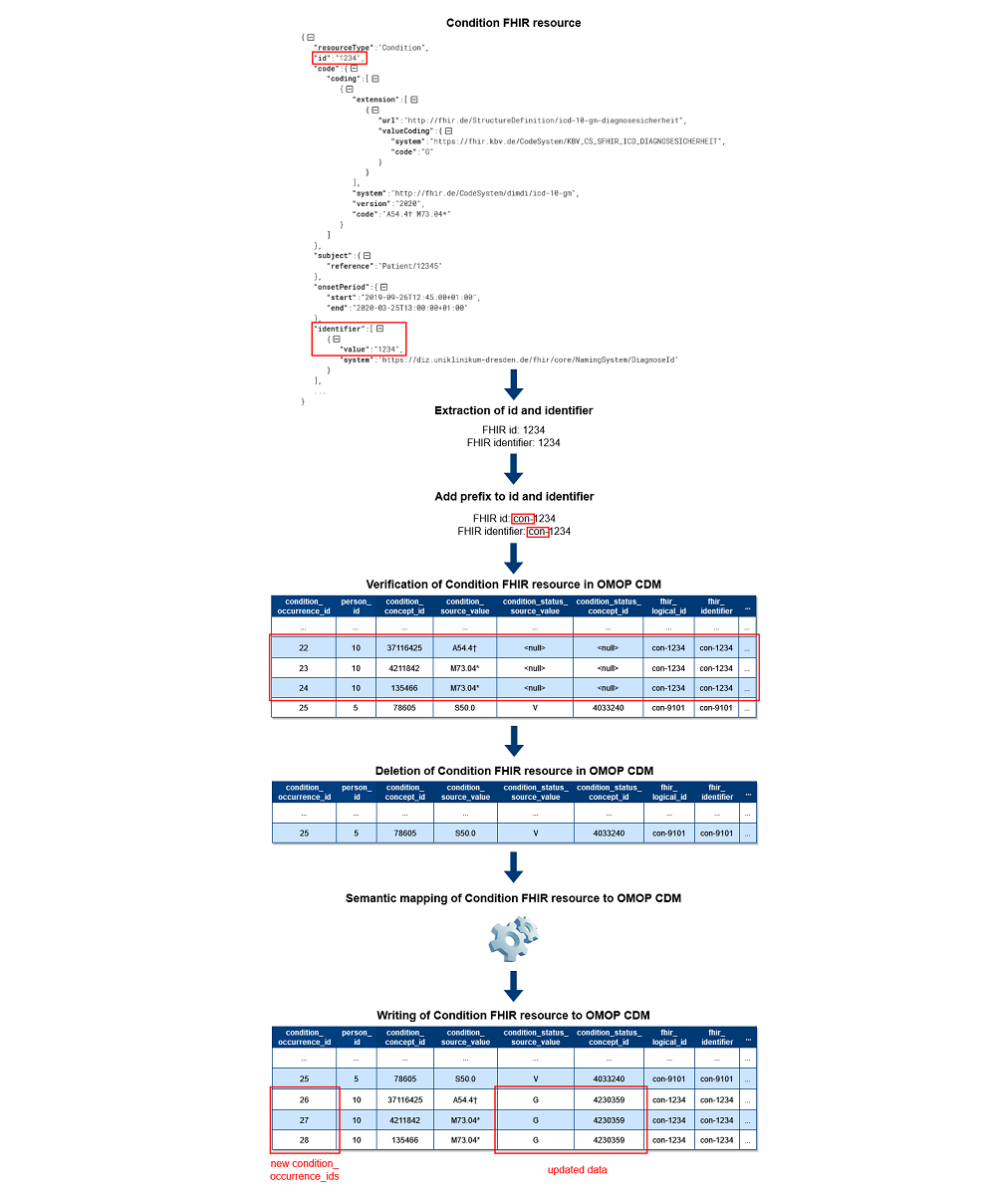
Excerpt of the data flow of the Condition Processor. CDM: Common Data Model; FHIR: Fast Healthcare Interoperability Resources; OMOP: Observational Medical Outcomes Partnership.

Based on the unique identification of FHIR resources in OMOP CDM, it is now possible to guarantee data correctness in OMOP CDM during incremental loading. Figure 1 shows the exemplary data flow for Condition FHIR resources for the second approach with two new columns. First, the Processor extracts the id and identifier used in FHIR. After that, the prefix is added to both values. Regardless of whether the data was created, updated, or deleted in the source, the ETL process next verifies each processed FHIR resource’s existence in OMOP CDM using the mapping tables or two new columns. During the verification, records are deleted in OMOP CDM if they were found. This approach is also used for updated FHIR resources to avoid incomplete updates for cross-domain mappings in OMOP CDM. Consequently, we do not perform updates on the existing records in OMOP CDM except Patient and Encounter FHIR resources to ensure referential integrity in OMOP CDM. In case FHIR resources are marked as deleted in the source, the processing is completed. Otherwise, the same semantic mapping logic as for bulk loading [2] applies afterward, and the data of the FHIR resources are written to OMOP CDM as new records with new OMOP ids.

### Evaluation of the Incremental Load Process

For the evaluation of the incremental load process, we defined and executed two ETL test designs. First, we tested which approach to store the mapping between id and identifier used in FHIR with the id used in OMOP CDM was the most performant. For this purpose, we implemented a separate ETL process version for each approach. Afterward, we executed the ETL process as bulk load first and as incremental load afterward, and compared the execution times between the mapping table approach and the column approach. For further evaluation of the incremental load process, we have chosen the most performant approach, resulting in a new optimized ETL process version for the second ETL test design.

To test the achievement of the 3 requirements identified during the initial analysis of our ETL process, we defined and executed a second ETL test design (Table 2) that compares the results of bulk loading with those of incremental loading regarding performance and data correctness. Our hypotheses here are that the execution time of incremental loading alone is less than bulk loading including daily updates and that the amount of data per table in OMOP CDM is identical after incremental loading and bulk loading, including daily updates.

**Table 2.** Extract-Transform-Load test design regarding performance and data correctness.

Test focus	Hypothesis
Performance	t(bulk loading (3 mon)) + t(incremental loading (1 d)) < t(bulk loading (3 mon)) + t(bulk loading (3 mon + 1 d))
Data correctness	#((bulk loading (3 mon)) + (incremental loading (1 d))) = #(bulk loading (3 mon + 1 d))

For both ETL test designs, we used a total of 3,802,121 synthetic FHIR resources version R4 based on the MI-I CDS version 1.0, which were generated using random values. Furthermore, we simulated CUD-FHIR resources for testing incremental loading for 1 day. For the simulation, we checked the frequency distribution of CUD data per domain in our source system with real-world data for 8 days and calculated the average value (see Multimedia Appendix 4). In addition, we set up one OMOP CDM v5.3.1 database as the target and executed the ETL process according to our test designs. For both ETL tests, we tracked the execution times based on the time stamps in the logging file of the ETL process until the corresponding job finished successfully. In a second step, we recorded the data quantity for each filled table in OMOP CDM and compared the results between the two ETL loading options for the second ETL test.

## Results

### Architecture of the ETL Process

**Figure 2.**
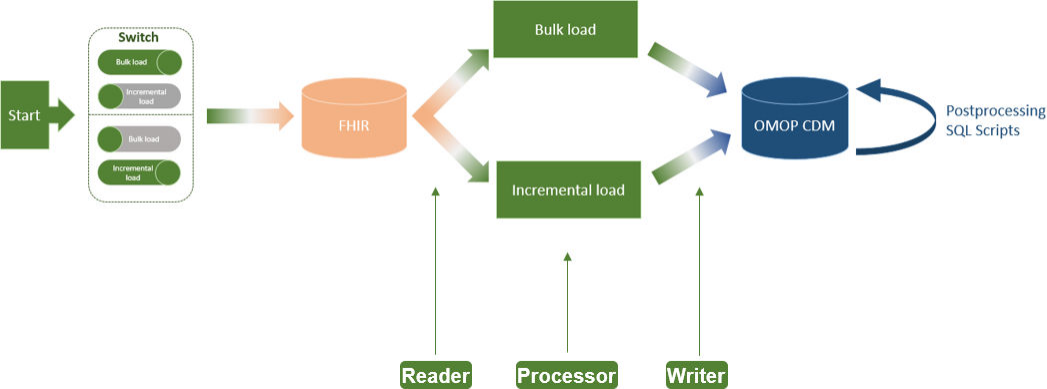
Architecture of the FHIR-to-OMOP Extract-Transform-Load process including incremental load. CDM: Common Data Model; FHIR: Fast Healthcare Interoperability Resources; OMOP: Observational Medical Outcomes Partnership.

The implemented ETL process extension for incremental loading of FHIR resources to OMOP CDM has not changed the basic architecture of the ETL process as proposed by Peng et al [2], consisting of Reader, Processor, and Writer (Figure 2). The only addition is a switch at the beginning of the ETL process, which allows the user to select between bulk load and incremental load (requirement A). Moreover, we configured the Reader for incremental loading of CUD-FHIR resources on a daily basis (requirement B). In the Processor, we added the logic of the verification of CUD-FHIR resources and their deletion from OMOP CDM if they already exist (requirement C).

The ETL process covering bulk and incremental load is available in the OHDSI repository ETL-German-FHIR-Core [25].

### Findings of the First ETL Test

The first ETL test focused on the performance measurement of the mapping table approach versus the column approach. First, we executed both ETL approaches as a bulk load. The column approach took about 30 minutes to transform FHIR resources to OMOP CDM. In contrast, the mapping table approach was still not finished after 4 hours. Therefore, we stopped the ETL execution and did not test the incremental loading anymore. Consequently, for the incremental ETL design, we decided to use the column approach due to its better performance and executed the subsequent performance evaluations with it.

### Findings of the Second ETL Test

The second ETL test dealt with testing our two hypotheses in Table 2. First, we compared the execution times between a bulk load (3 mon + 1 d) and an initial bulk load (3 mon) followed by an incremental load (1 d). For this, each loading option was executed three times. Based on the results, we calculated the average execution times. The performance results (Multimedia Appendix 5) show that an initial bulk load (13.31 min) followed by a daily incremental load (2.12 min) is more efficient than an everyday full load (17.07 min). Looking at the percentage improvement in performance, it can be shown that incremental loading had 87.5% less execution time than a daily full load (2.12 min compared to 17.07 min). Referring to our first hypothesis, we were able to prove our initial assumption.

After the execution of both loading options, we further checked the data quantity for each filled table in OMOP CDM and compared the results of it. As shown in Table 3, both loading options resulted in the same amount of data (Multimedia Appendix 5). Consequently, we were also able to confirm our second hypothesis regarding data correctness in OMOP CDM.

**Table 3.** Results of the data quantity comparison in the Observational Medical Outcomes Partnership Common Data Model (OMOP CDM) between bulk and incremental load.

	Bulk load (3 mon + 1 d), n	Bulk load (3 mon) + incremental load (1 d), n
Care_site	152	152
Condition_occurrence	800,640	800,640
Death	857	857
Drug_exposure	1,171,521	1,171,521
Fact_relationship	2,323,894	2,323,894
Measurement	231,369	231,369
Observation	511,844	511,844
Observation_period	15,037	15,037
Person	15,037	15,037
Procedure_occurrence	168,384	168,384
Source_to_concept_map	251	251
Visit_detail	43,929	43,929
Visit_occurrence	29,898	29,898

## Discussion

### Principal Findings

Based on the partial results for research questions 1 and 2, we defined three methods to integrate incremental loading into our ETL process design. In this context, the 3 identified requirements from the initial analysis could be implemented by taking existing approaches from the literature into account (research question 3). Moreover, the incremental load process was tested at 10 university hospitals in Germany and ensures daily data transfer to OMOP CDM for the CTRSS. This proves that the ETL process is also suitable for real-world data, although it was developed with synthetic data.

Currently, our ETL process requires FHIR resources following the MI-I core data set specification. However, our initial requirements analysis showed that the implemented incremental ETL logic does not affect the semantic mapping from FHIR to OMOP CDM described by Peng et al [2]. In consequence, the incremental ETL logic is independent of the data available in the FHIR format. Therefore, it can be used to incrementally transform international FHIR profiles such as the US Core Profiles [26] to OMOP CDM.

### Limitations

Nevertheless, the ETL process has limitations in its execution capabilities. As our FHIR resources comprise a logical id corresponding to the id in our source system, our ETL process is currently not able to deal with changing server end points, resulting in changing logical ids. Additionally, so far, we have not included an option to automatically start incremental loading nor do we support real-time streaming (eg, via Apache Kafka [27]). These limitations are part of future work.

To evaluate incremental loading compared to bulk loading, we performed two ETL tests (research question 4). The results of the performance tests showed that the column approach is more performant than the mapping table approach. Our suspected explanation for this is that the mapping table approach requires additional tables to be filled besides the standardized tables in OMOP CDM. Consequently, during the verification of FHIR resources in OMOP CDM, a lookup and deletion in several tables (mapping tables and standardized tables) is necessary, whereas the column approach only accesses the standardized tables.

Referring to our two hypotheses regarding performance and data correctness between incremental loading and bulk loading, we showed that our initial assumptions were proven. With the option of an incremental ETL process, we were able to reduce execution times to provide data in OMOP CDM on a daily basis, without data loss compared to the bulk load ETL process. For our future work, we want to further evaluate at what point bulk load is more worthwhile than incremental loading. The results of these evaluations will be incorporated into the automation concept.

During the productive use of the ETL process, we identified two issues that have to be considered in the context of incremental loading to OMOP CDM. First, OHDSI provides a wide range of open-source tools (eg, ATLAS [28]) for cohort definitions or statistical analyses. To make ATLAS work on the data in OMOP CDM, a summary report has to be generated in advance using ACHILLES [29] (Automated Characterization of Health Information at Large-Scale Longitudinal Evidence Systems), an R package that provides characterization and visualization. Regarding the incremental ETL process, ACHILLES has to be run after each successful execution of the incremental ETL process.

A second issue that needs to be addressed relates to the ids in the standardized tables in OMOP CDM. The incremental loading process requires the assignment of new ids in the OMOP CDM. While this was not a problem during development, it becomes obvious when a large amount of data is processed. In this context, the maximum id in the tables of OMOP CDM was reached, which led to a failure of the ETL process. We need to pay special attention to this point and find a solution (eg, by reusing deleted ids or by changing the ETL process in a real updating ETL process). As this problem does not occur during the bulk load process, a current workaround is to start that process if the incremental load fails, which is possible as our process comprises bulk and incremental load options.

### Conclusions

The presented ETL process from FHIR to OMOP CDM now enables both bulk and incremental loading. To receive daily updated recruitment proposals with the CTRSS, the ETL process no longer needs to be executed as a bulk load every day. One initial load supplemented by incremental loads per day meets the requirements of the CTRSS while being more performant. Moreover, since the incremental ETL logic is not restricted to the MI-I CDS specification, it can also be used for international studies that require daily updated data from FHIR resources in OMOP CDM. To be able to use not only the logic of incremental loading internationally, but the whole ETL process itself, the support of arbitrary FHIR profiles is needed. This requires a modularization and generalization of current ETL processes. For that, we will evaluate the extension to metadata-driven ETL in the near future.

## Supplementary material

10.2196/47310 Multimedia Appendix 1 Results and screenings of the literature review on July 14, 2021.

10.2196/47310 Multimedia Appendix 2 Results and screenings of the literature review on November 28, 2022.

10.2196/47310 Multimedia Appendix 3 Results and screenings of the literature review on February 22, 2023.

10.2196/47310 Multimedia Appendix 4 Frequency distribution of created, updated, and deleted data.

10.2196/47310 Multimedia Appendix 5 Results of the second Extract-Transform-Load test.

## Abbreviations

ACHILLES: Automated Characterization of Health Information at Large-Scale Longitudinal Evidence Systems
CDC: change data capture
CDM: Common Data Model
CDS: Core Data Set
CTRSS: clinical trial recruitment support system
CUD: created, updated, or deleted
ETL: Extract-Transform-Load
FHIR: Fast Healthcare Interoperability Resources
HL7: Health Level Seven
MI-I: Medical Informatics Initiative Germany
MIRACUM: Medical Informatics in Research and Care in University Medicine
OHDSI: Observational Health Data Sciences and Informatics
OMOP: Observational Medical Outcomes Partnership
